# Supplementary material for: Identification of a c-type heme oxygenase and its function during acclimation of cyanobacteria to nitrogen fluctuations
Source: Commun Biol. 2023 Sep 15;6:944. doi: 10.1038/s42003-023-05315-x (PMC10504260; doi:10.1038/s42003-023-05315-x)
Supplement: Supplementary file 2 — Supplementary information [file 42003_2023_5315_MOESM2_ESM.pdf]

Supplementary information

Identification of a *c*-type heme oxygenase and its function during acclimation of cyanobacteria to nitrogen fluctuations

Zhaoxing Ran[1,2], Zhenyu Du[2], Gengkai Miao[2], Mei Zheng[2], Ligang Luo[2], Xiaoqin Pang[2], Lanzhen Wei[2], Dezhi Li[1,3,4,5], Weimin Ma[2]

[1]School of Ecological and Environmental Sciences, East China Normal University, Shanghai 200241, China.
[2]College of Life Sciences, Shanghai Normal University, Shanghai 200234, China.
[3]Key Laboratory of Urbanization and Ecological Restoration of Shanghai, Shanghai 200241, China.
[4]Institute of Eco-Chongming (IEC), 20 Cuiniao Rd, Chenjia Zhen, Chongming, Shanghai, 202162, China
[5]Technology Innovation Center for Land Spatial Eco-restoration in Metropolitan Area, Ministry of Natural Resources, 3663 N. Zhongshan Road, Shanghai, 200062, China

Correspondence and requests for materials should be addressed to W.M. (email: wma@shnu.edu.cn) or to D.L. (email: dzli@des.ecnu.edu.cn) or to L.W. (email: weilz@shnu.edu.cn)

This PDF file includes:
Supplementary Figs. 1 to 21
Supplementary Table 1

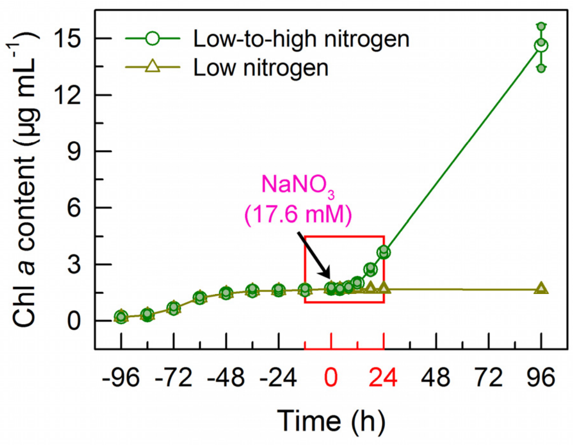

Supplementary Fig. 1 | Change in chlorophyll *a* content after the nitrate is added to chlorotic cells. The nitrate is added to the cells grown in low-nitrate medium or without and the time points in red box were extensively analyzed in this study. Error bars denote the standard deviations of three independent measurements (n = 3). Chl *a*, chlorophyll *a*.

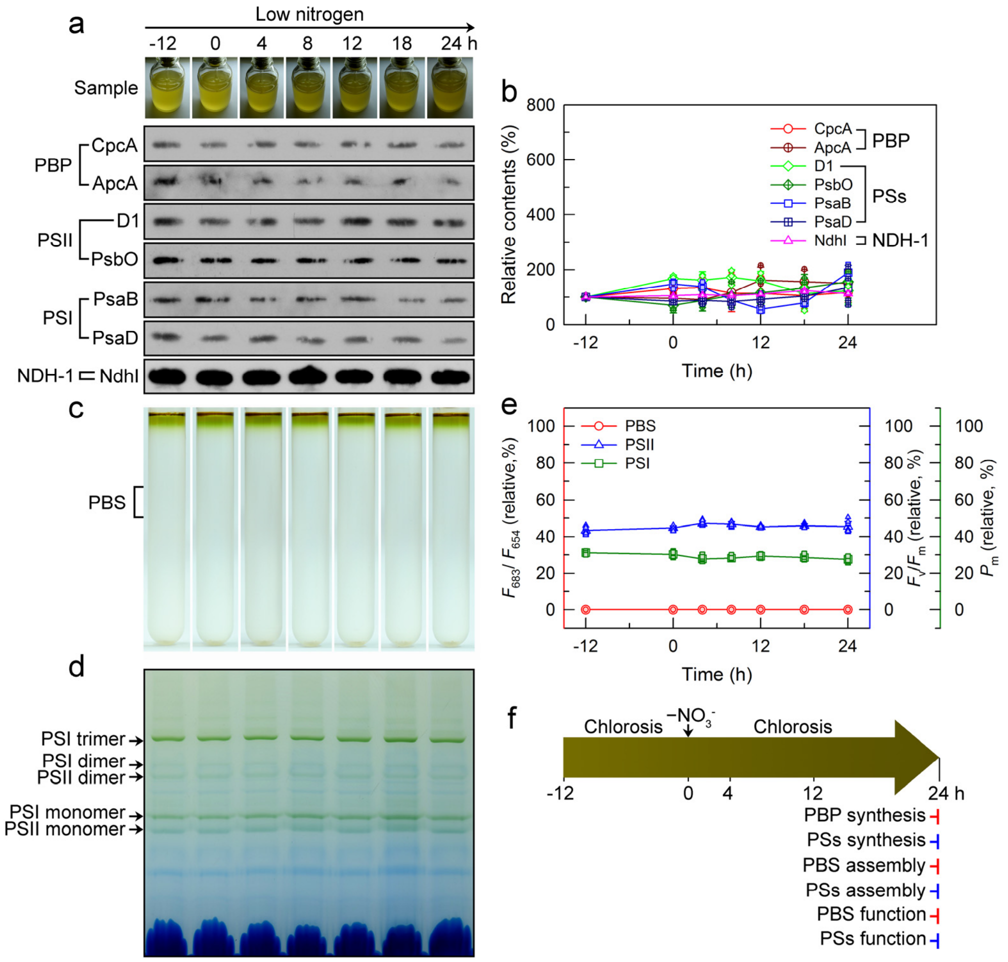

Supplementary Fig. 2 | Synthesis, assembly and function of phycobiliprotein and both photosystems under continuous low nitrogen conditions. a, b Time-course synthesis profiles of phycobiliprotein (PBP) and both photosystems (PSs) (**a**) and their quantification using ImageJ (**b**) under continuous low nitrogen conditions. Total protein corresponding to 3×10^7 cells was loaded onto each lane and NdhI was used as a sample loading control. Error bars denote the standard deviations of three independent measurements (n = 3) for CpcA, ApcA, D1, PsbO, PsaB and NdhI and of four independent measurements (n = 4) for PsaD. **c, d** Time-course assembly profiles of phycobilisome (PBS) isolated by sucrose density gradient centrifugation (**c**) and PSs isolated by BN-PAGE (**d**) under continuous low nitrogen conditions. **e** Time-course function profiles of PBS and PSs under continuous low nitrogen conditions. Red lines (circle): PBS (n = 6); blue lines (triangle up): PSII (n = 9); green lines (square): PSI (n = 9). **f** A model schematically represents the temporal sequence of synthesis, assembly and function of PBP and PSs under continuous low nitrogen conditions.

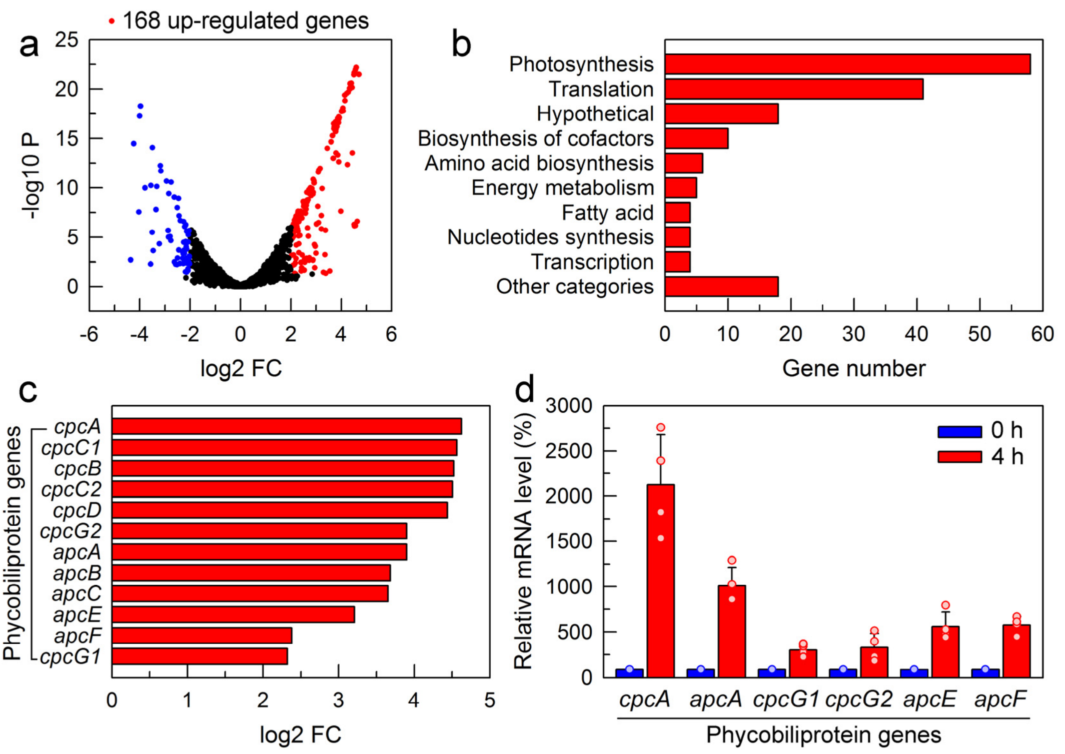

Supplementary Fig. 3 | A comparative transcriptomic analysis reveals up-regulation of many apo-phycobiliprotein-related genes after the nitrate was added to chlorotic cells. a Volcano plot indicated that the 168 genes were up-regulated after the nitrate was added to chlorotic cells for 4 h (see red dots). $n = 3$. **b** Gene Ontology classification of 168 up-regulated genes was performed based on function annotation in CyanoBase database. **c** Fold change of apo-phycobiliprotein genes after the nitrate was added to chlorotic cells for 4 h. **d** RT-qPCR analysis confirmed the transcriptomic data of apo-phycobiliprotein genes after the nitrate was added to chlorotic cells for 4 h. Error bars denote the standard deviations of four independent measurements ($n = 4$).

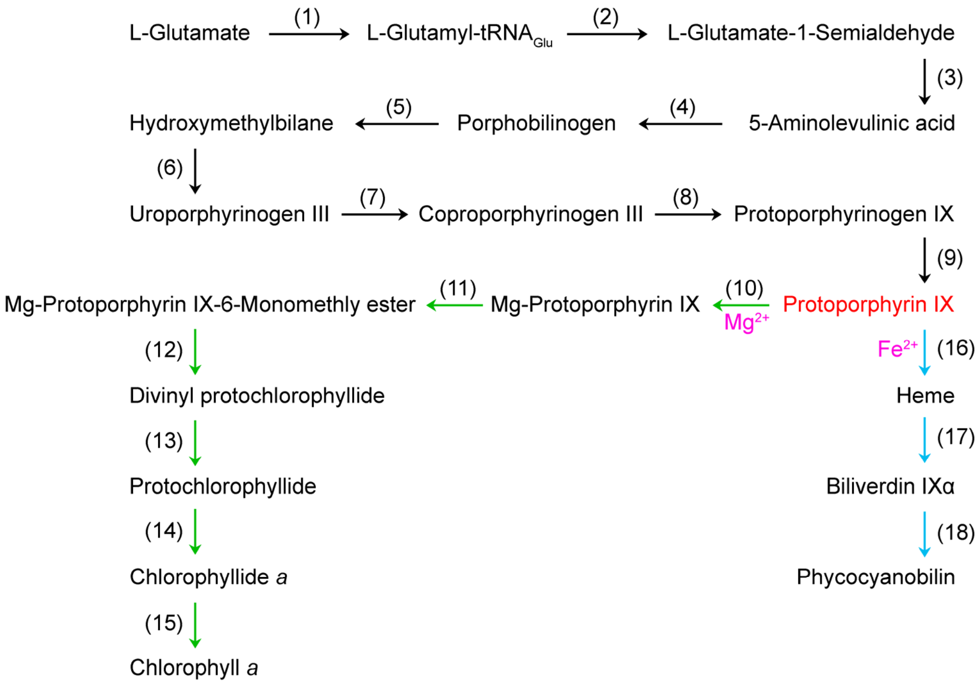

(1) Glutamyl-tRNA synthetase (2) Glutamyl-tRNA reductase

(3) Glutamate-1-semialdehyde aminomutase (4) Porphobilinogen synthase

(5) Hydroxymethylbilane synthase (6) Uroporphyrinogen III synthase

(7) Uroporphyrinogen III decarboxylase (8) Coproporphyrinogen III oxidase

(9) Protoporphyrinogen IX oxidase (10) Mg-chelatase

(11) S-adenosyl-L-methionine: Mg-protoporphyrin IX methyltransferase

(12) Mg-protoporphyrin IX monomethyl ester oxidative cyclase

(13) Divinyl-(proto) chlorophyllide reductase (14) Protochlorophyllide oxidoreductase

(15) Chlorophyll synthase (16) Fe-chelatase (17) Heme oxygenase

(18) Phycocyanobilin: Fd oxidoreductase

Supplementary Fig. 4 | Cyanobacterial tetrapyrrole biosynthetic pathway. In cyanobacteria, the tetrapyrrole biosynthetic pathway begins with L-glutamate to protoporphyrin IX (black arrows) and is branched into phycocyanobilin (blue arrows) and chlorophyll *a* (green arrows) biosynthetic lines.

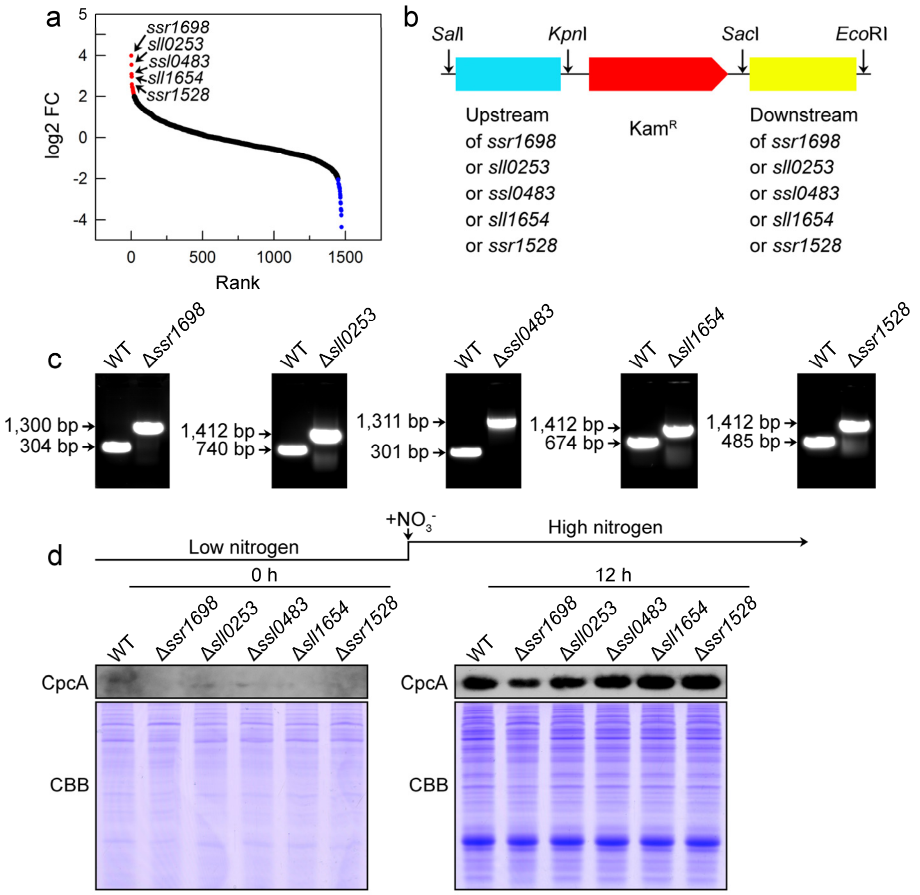

Supplementary Fig. 5 | Screening and identification of an unknown gene *ssr1698* that is involved in the phycobiliprotein synthesis. a The top five up-regulated unknown genes in our transcriptomic data were selected as candidates for phycobiliprotein synthesis. **b** Construction of plasmids used to generate the inactivation mutants of the top five up-regulated unknown genes (Δ*ssr1698*, Δ*sll0253*, Δ*ssl0483*, Δ*sll1654* and Δ*ssr1528*). **c** PCR segregation analyses of these five mutants using their respective specific primers (Supplementary Table 1). **d** Coomassie Brilliant Blue (CBB) staining profiles of total proteins from the wild-type (WT) and these five mutants strains after the nitrate was added to chlorotic cells for 12 h (right side) or not (left side) and their immunoblotting using the antibody against phycobiliprotein CpcA. Total protein corresponding to 3×10^6 cells was loaded onto each lane.

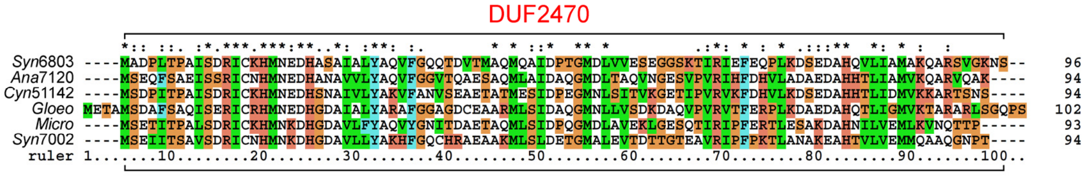

Supplementary Fig. 6 | Sequence alignment of Ssr1698 of *Synechocystis* 6803 and its homologues from other species. The sequence of Ssr1698 from *Synechocystis* sp. PCC 6803 (*Syn*6803) was compared with homologous sequences from *Anabaena* sp. PCC 7120 (*Ana*7120), *Cyanothece* sp. ATCC 51142 (*Cyn*51142), *Gloeobacter violaceus* PCC 7421 (*Gloeo*), *Microcystis aeruginosa* NIES-843 (Micro) and *Synechococcus* sp. PCC 7002 (*Syn*7002). The DUF2470 domain analysis was performed by the NCBI Web service (https://www.ncbi.nlm.nih.gov/). The sequences were aligned using ClustalX 1.83. Asterisks indicate identical amino acids; colons and dots indicate conserved amino acid substitutions.

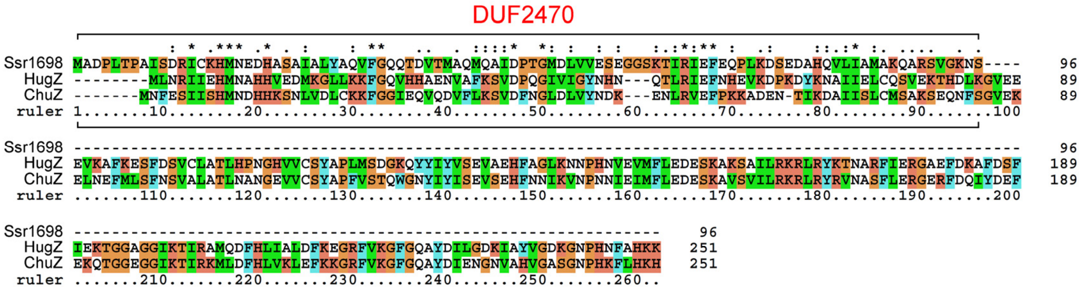

Supplementary Fig. 7 | The DUF2470 domain of cyanobacterial Ssr1698 is also identified in bacterial HugZ and ChuZ. The sequence of Ssr1698 from *Synechocystis* sp. PCC 6803 was aligned with HugZ and ChuZ sequences from *Helicobacter pylori* and *Campylobacter jejuni*, respectively. The DUF2470 domain analysis was performed by the NCBI Web service (https://www.ncbi.nlm.nih.gov/).

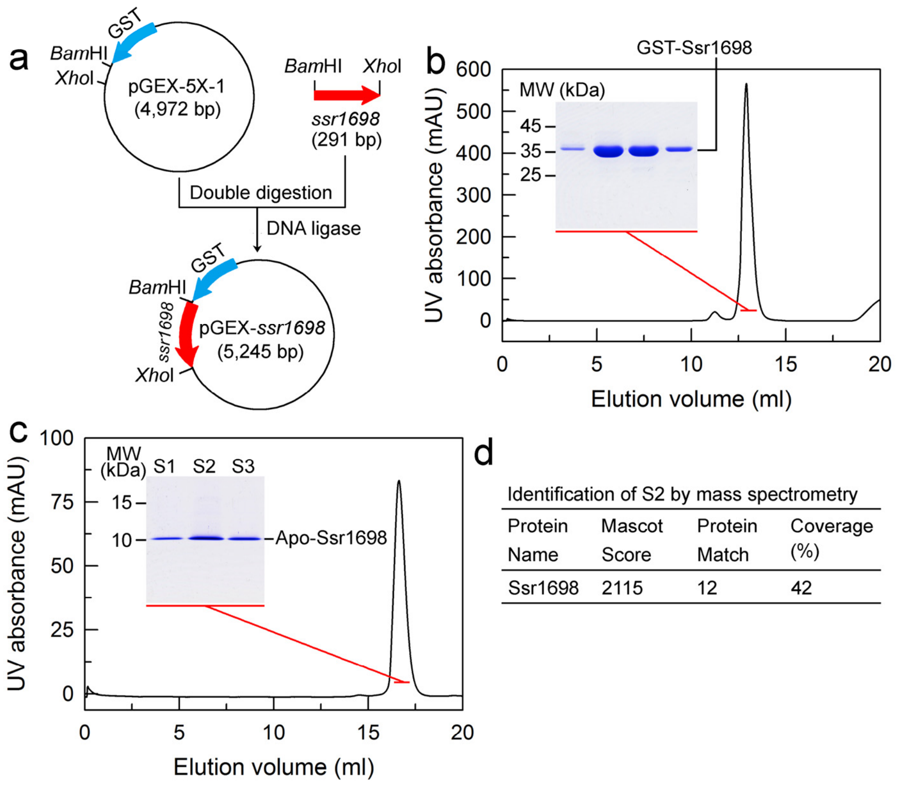

Identification of S2 by mass spectrometry

Protein Name	Mascot Score	Protein Match	Coverage (%)
Ssr1698	2115	12	42

Supplementary Fig. 8 | Expression and purification of Ssr1698 protein. a Construction of pGEX-5X-1 vector to generate the pGEX-*ssr1698* expression plasmid. **b** Purification of GST-Ssr1698 using GST affinity chromatography followed by size-exclusion chromatography. Position of GST-Ssr1698 in the SDS-PAGE gel is indicated. **c** Purification of tag-less Ssr1698 using size-exclusion chromatography after purified GST-Ssr1698 is cleaved by Factor Xa protease. Sample 1 (S1), S2 and S3 are analyzed by the SDS-PAGE gel and the position of tag-less Ssr1698 (Apo-Ssr1698) in the SDS-PAGE gel is indicated. **d** Identity of Ssr1698 in S2 is confirmed by mass spectrometry.

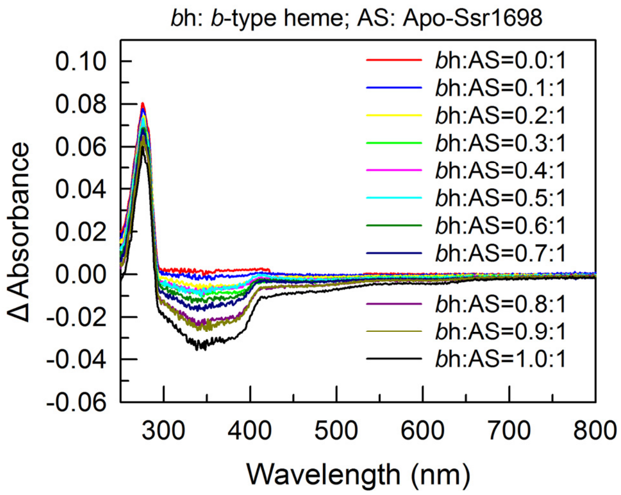

Supplementary Fig. 9 | Apo-Ssr1698 does not specifically bind to *b*-type heme. The hemin (*b*-type heme) titration of apo-Ssr1698 (20 μM) as monitored by the difference absorption spectra in PBS buffer (pH 7.4) and 20 μM BSA. The absence of positive characteristic absorption peaks indicates that apo-Ssr1698 does not specifically bind to *b*-type heme.

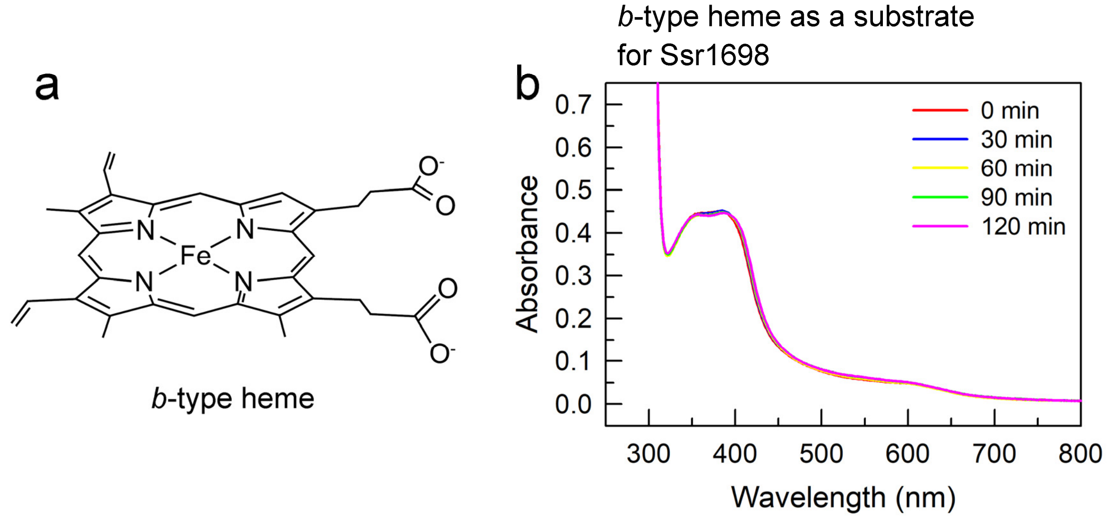

Supplementary Fig. 10 | The degradation assays of *b*-type heme with Ssr1698. a Chemical structure of *b*-type heme. **b** The data of characteristic Soret peak of *b*-type heme around 400 nm and its shoulder peaks between 500 nm and 600 nm indicate that Ssr1698 lacked any observable reactivity with *b*-type heme.

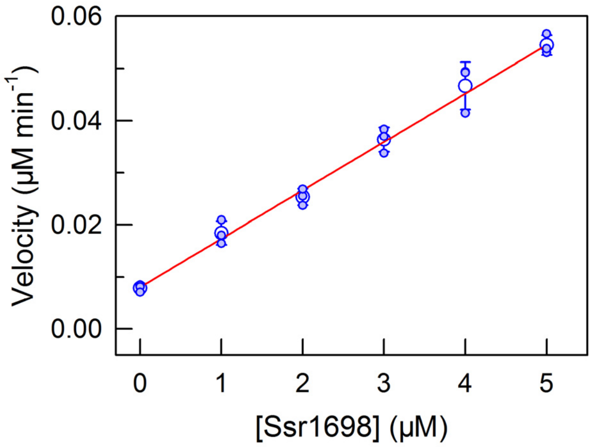

Supplementary Fig. 11 | The degradation rate of *c*-type heme catalyzed by different Ssr1698 concentrations. Comparison of the rates of degradation of 9 μM MP-11 (*c*-type heme) in the presence of 0 to 5 μM Ssr1698 and 2 μM catalase. Error bars denote the standard deviations of three independent measurements ($n = 3$).

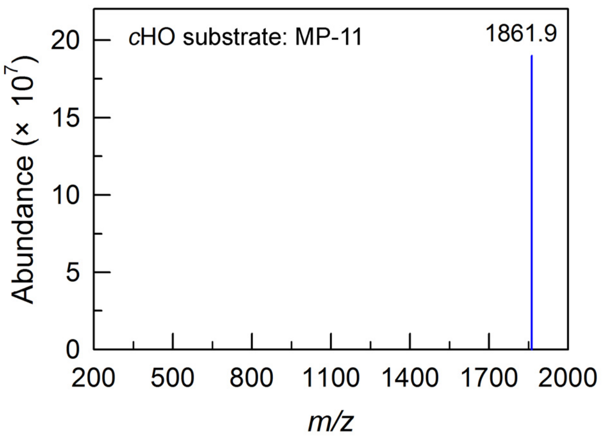

Supplementary Fig. 12 | Liquid chromatograph-mass spectrometer (LC-MS) analysis of MP-11, a substrate for the *c*-type heme oxygenase (*c*HO). Simplified LC/MS spectrum of the *c*HO substrate MP-11 showed that the peptide was not cleaved by the LC-MS condition.

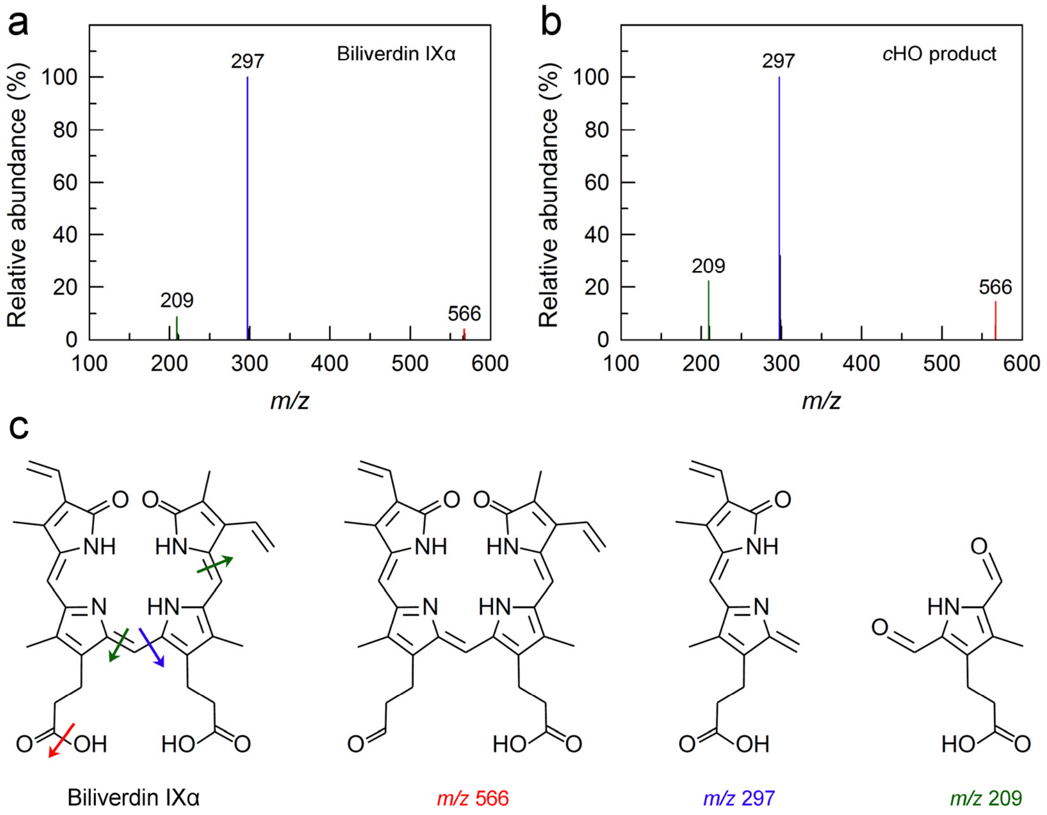

Supplementary Fig. 13 | The analysis of mass spectrometry fragmentation pattern of the 583.3 *m/z* peak of Ssr1698 reaction with MP-11. a Fragmentation profiles of biliverdin IXα, a standard reference sample. **b** Fragmentation profiles of 583.3 *m/z* peak (see Fig. 4c) of Ssr1698 reaction with MP-11. **c** The arrows of different colors indicate the mass spectrometry fragmentation patterns.

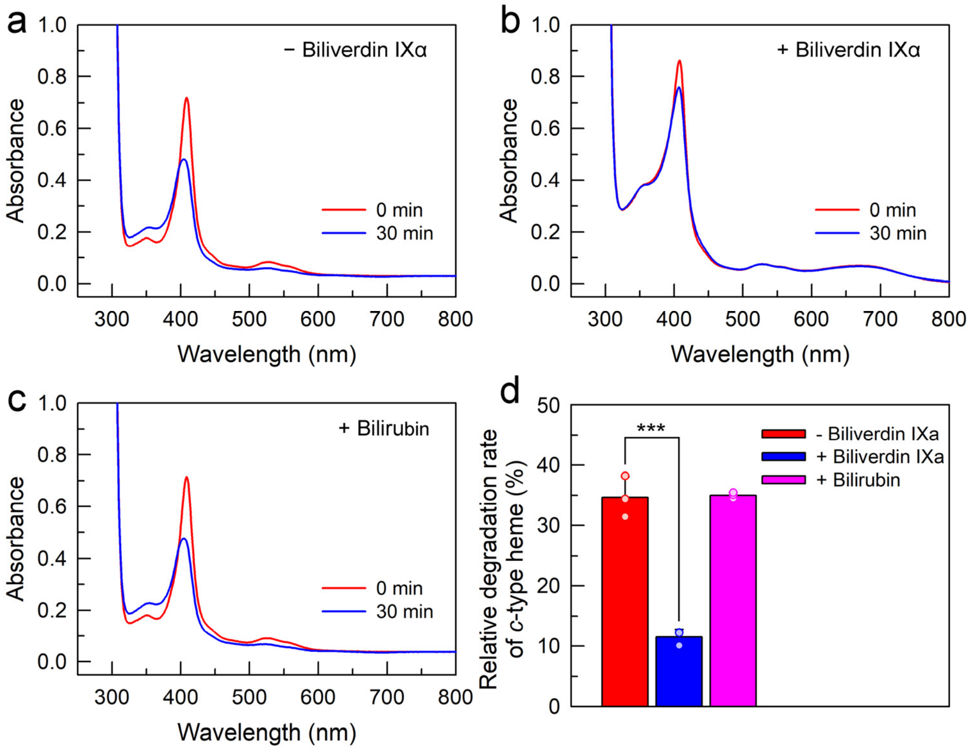

Supplementary Fig. 14 | The activity of Ssr1698 as *c*-type heme oxygenase is suppressed by its product biliverdin IXα. a-c The degradation assay of *c*-type heme catalyzed by Ssr1698 after biliverdin IXα (**b**) and bilirubin (**c**) are added to the reaction system or without (**a**). **d** Relative degradation rate of *c*-type heme. The peak D-value of Soret band at 0 and 30 min is compared in the presence of biliverdin IXα (**b**) and bilirubin (**c**) or not (**a**). Error bars denote the standard deviations of three independent measurements ($n = 3$); *** represents $P < 0.001$ ($P = 0.0004$).

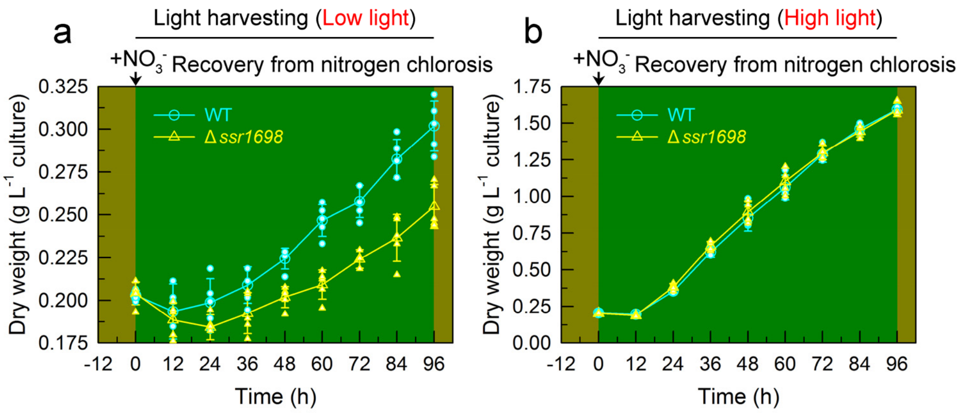

Supplementary Fig. 15 | The function of *c*HO-dependent phycobiliprotein under different light intensities during recovery from nitrogen chlorosis. a, b After the nitrate is added to the nitrogen-starved cells, the *c*HO-dependent phycobiliprotein is of great important for the growth of cells under low light (**a**, $n = 5$) and is redundant for the growth of cells under high light (**b**, $n = 4$).

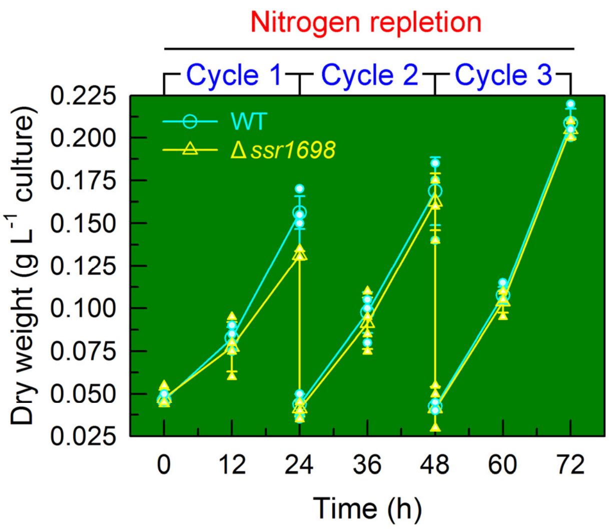

Supplementary Fig. 16 | The function of *c*HO-dependent phycobiliprotein in repeated cycles of nitrogen replete during recovery of growth light-illuminated cyanobacteria from nitrogen chlorosis. After the nitrate is added to the nitrogen-starved cells, the *c*HO-dependent phycobiliprotein becomes less important for the growth of cells in repeated cycles of nitrogen replete. Error bars denote the standard deviations of four independent measurements ($n = 4$).

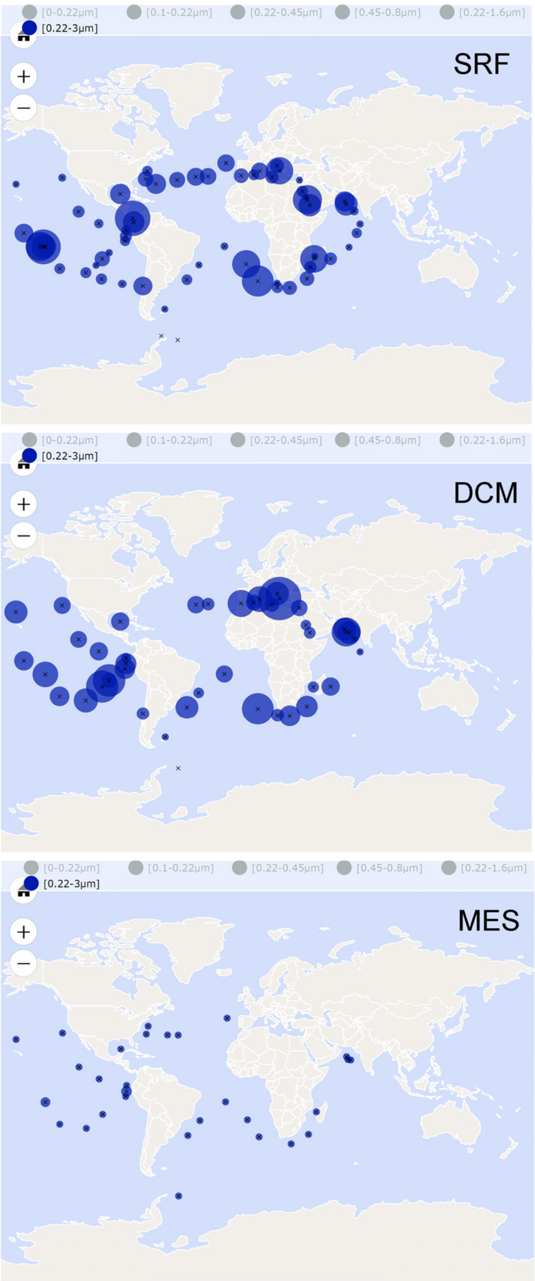

Supplementary Fig. 17 | Geographic distribution of Ssr1698 homolog sequences found in the Ocean Gene Atlas webserver. Geographic distribution and abundance of Ssr1698 homologs in surface water (SRF; upper panel), deep chlorophyll maximum layer (DCM; middle panel) and mesopelagic zone (MES; lower panel). The size of the filled circles is proportional to the abundance of the hits in one location compared to the total number of hits at the different sampled depths.

a

```
>ssr1698 hypothetical protein
          Length = 96

 Score = 38.1 bits (87), Expect = 6e-05,   Method: Compositional matrix adjust.
 Identities = 21/77 (27%), Positives = 36/77 (46%), Gaps = 3/77 (3%)

Query: 3  NRIIEHMNAHHVEDMKGLLKKFGQVHHAENVAFKSVDSQG---IVIGYNNNQTLRIEFNH 59
          +RI +HMN  H   +    + FGQ         +++D  G   +V     ++T+RIEF
Sbjct: 11 DRICKHMNEDHASAIALYAQVFGQQTDVTMAQMQAIDPTGMDLVVESEGGSKTIRIEFEQ 70

Query: 60 EVKDPKDYKNATIELCQ 76
           +KD +D     I + +
Sbjct: 71 PLKDSEDAHQVLIAMAK 87
```

b

```
>ssr1698 hypothetical protein
          Length = 96

 Score = 34.7 bits (78), Expect = 9e-04,   Method: Compositional matrix adjust.
 Identities = 23/82 (28%), Positives = 41/82 (50%), Gaps = 6/82 (7%)

Query: 4  ESIISHMNDHHKSNLVDLCKKFGGIEQVQDVFLKSVDFNGLDLVYNDK---ENLRVEF-- 58
          + I  HMN+ H S +    + FG    V    ++++D  G+DLV   +   + +R+EF
Sbjct: 11 DRICKHMNEDHASAIALYAQVFGQQTDVTMAQMQAIDPTGMDLVVESEGGSKTIRIEFEQ 70

Query: 59 PKKADENTIKDAIISLCMSAKS 80
          P K  E+     +I++   A+S
Sbjct: 71 PLKDSEDA-HQVLIAMAKQARS 91
```

Supplementary Fig. 18 | Short stretches of Ssr1698 share limited sequence similarity with HugZ or ChuZ. a, b Original CyanoBase BLASTP hit for Ssr1698 was obtained using the complete sequence of HugZ (**a**) or ChuZ (**b**).

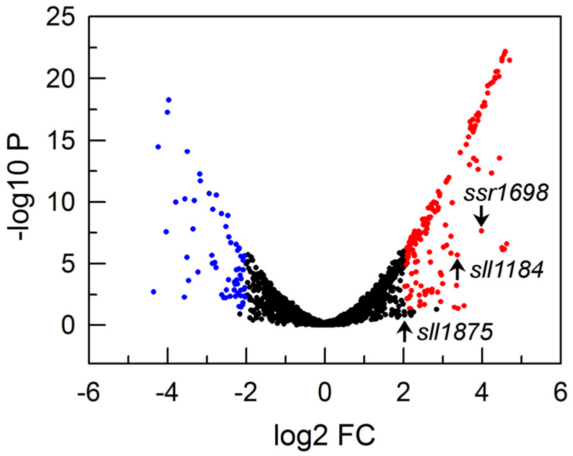

Supplementary Fig. 19 | Transcriptomic analysis reveals the differential levels of heme oxygenase genes after the nitrate was added to chlorotic cells for 4 h or not. Among them, *sll1184* and *sll1875* genes encode *b*-type heme oxygenases, *b*HO-1 and *b*HO-2, respectively, whilst *ssr1698* gene encodes *c*-type heme oxygenase, *c*HO.

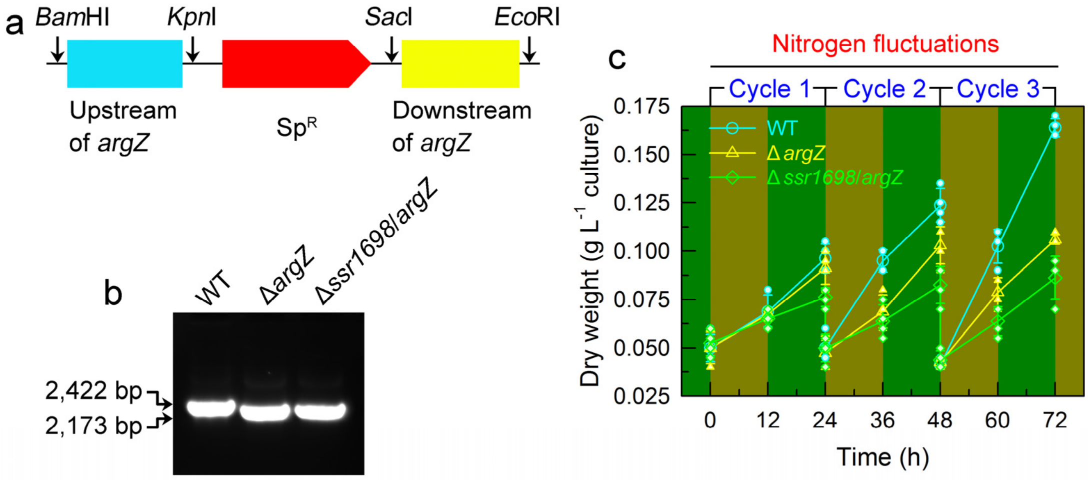

Supplementary Fig. 20 | The *c*HO-dependent phycobiliprotein and cyanophycin jointly function as nitrogen reservoirs. a Construction of plasmids used to generate the *argZ* inactivation mutant (Δ*argZ*). **b** PCR segregation analysis of the Δ*argZ* and Δ*ssr1698*/*argZ* mutants using the *argZ*-G and *argZ*-H primers (Supplementary Table 1). **c** The *c*HO-dependent phycobiliprotein and cyanophycin are of great important and are not redundant for cell growth during acclimation of cyanobacterial cells to a nitrogen-fluctuating environment. Error bars denote the standard deviations of four independent measurements ($n = 4$).

Supplementary Fig. 21 | Original uncropped blot/gel images.

Fig. 1a

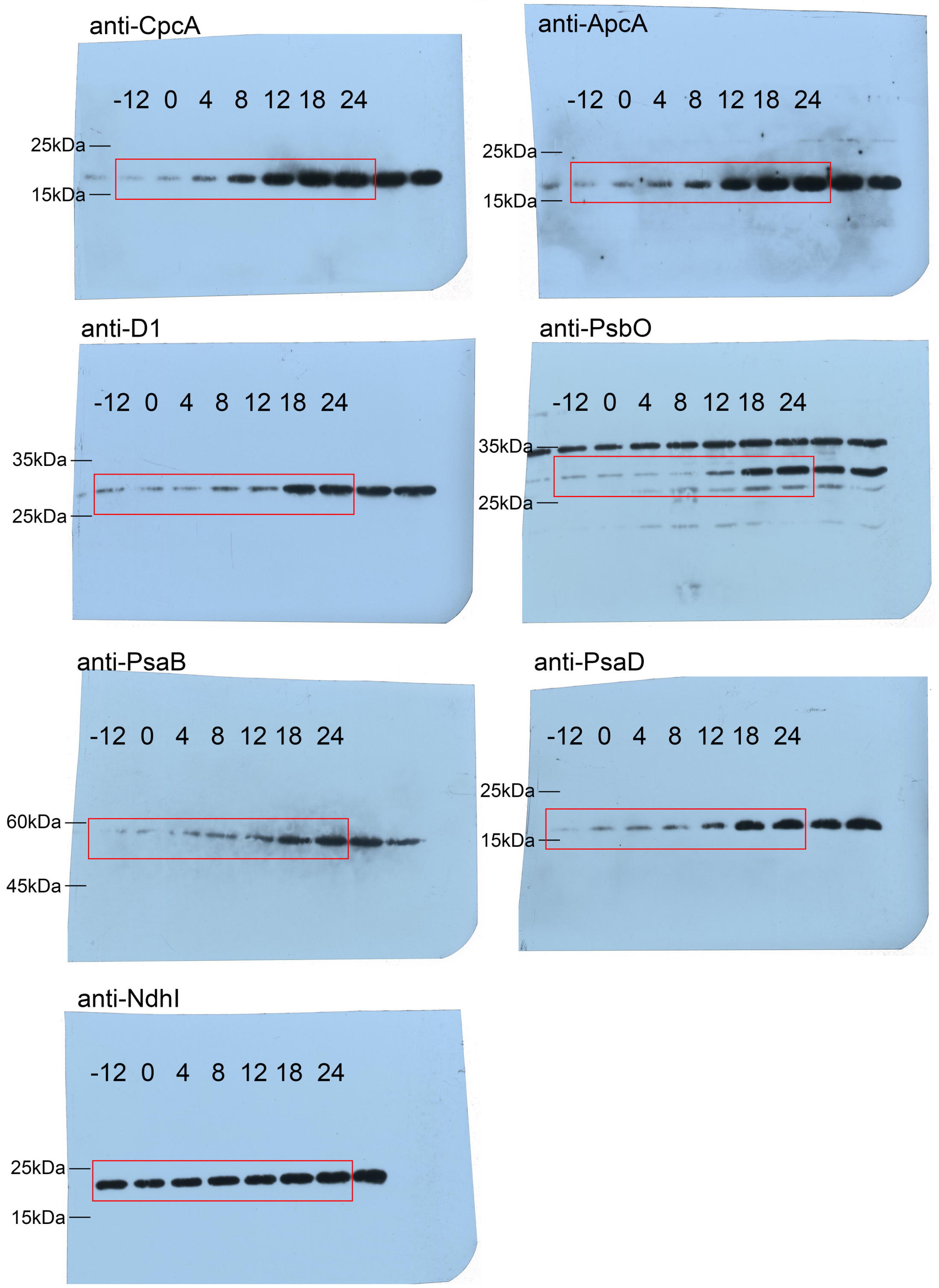

Fig. 1d

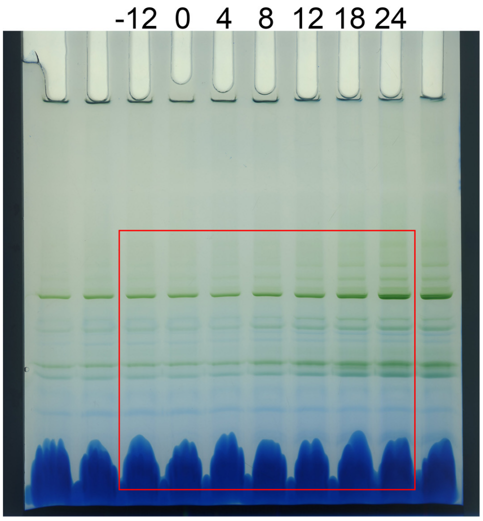

Fig. 2c

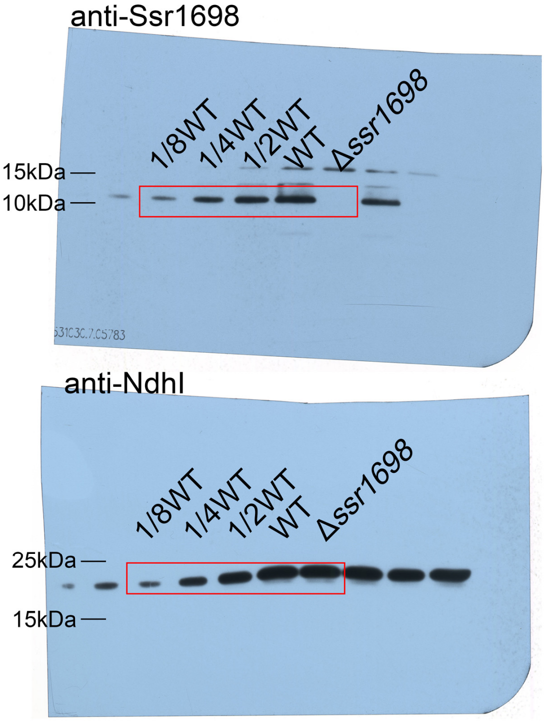

Fig. 2d

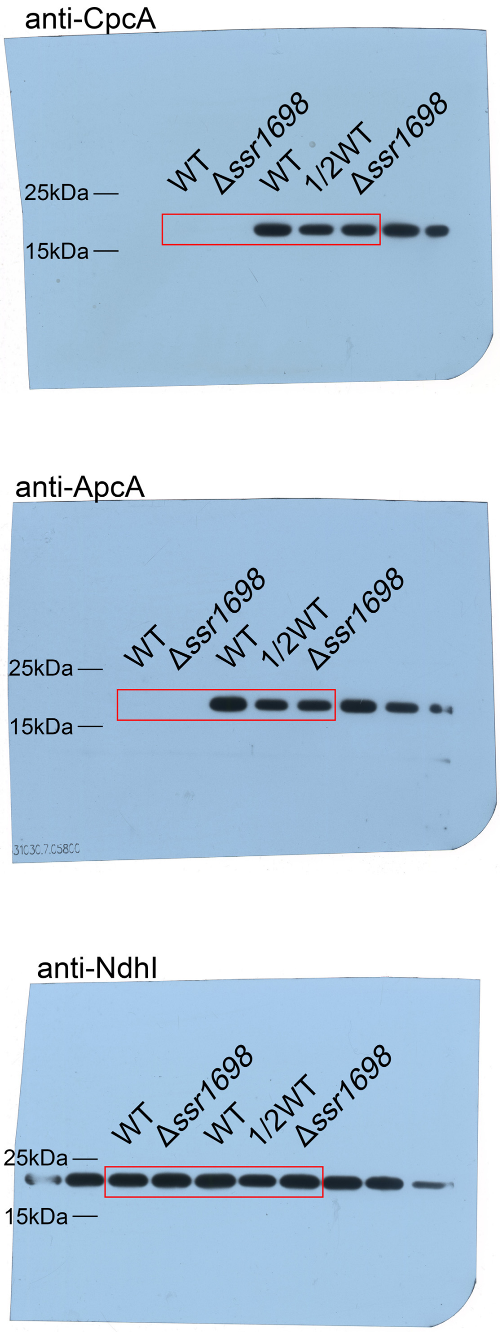

Fig. 3d

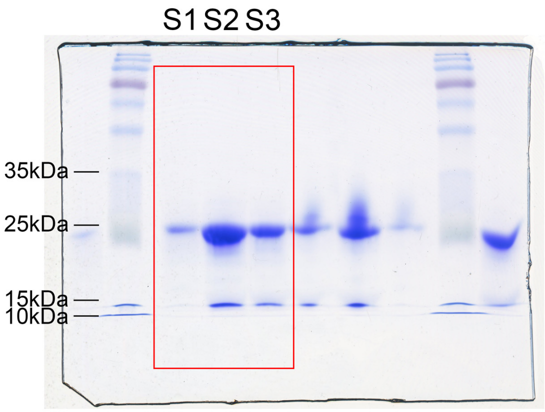

Supplementary Fig. 2d

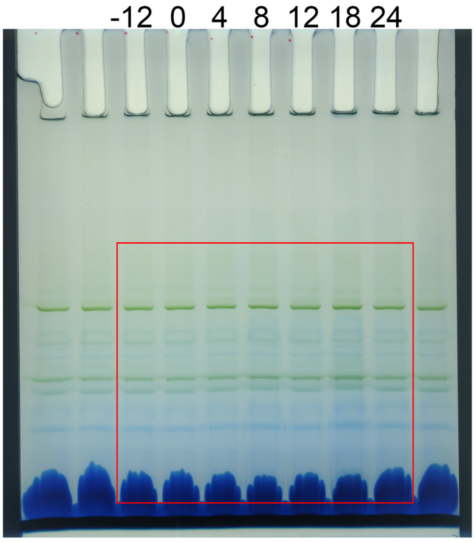

Supplementary Fig. 2a

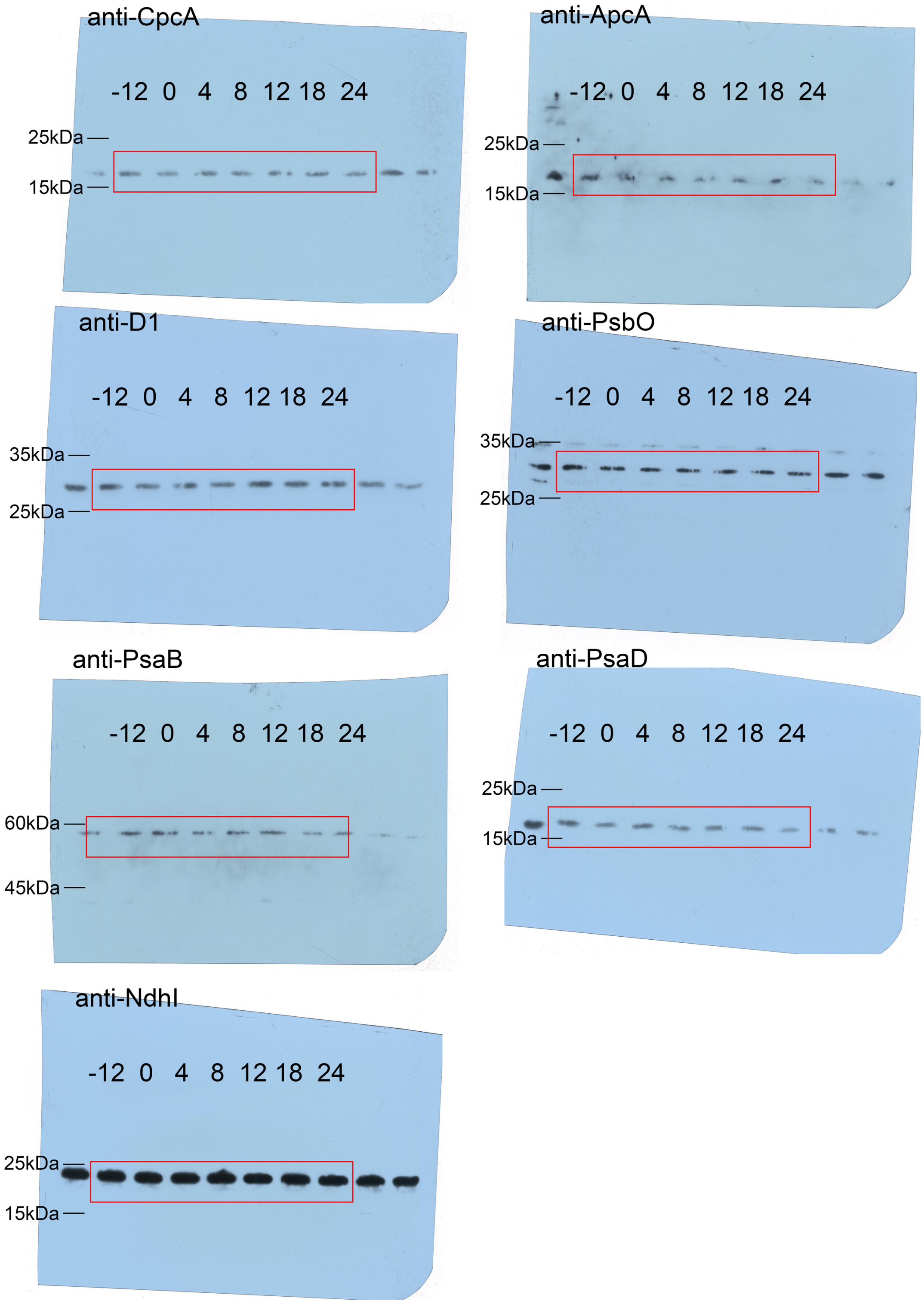

Supplementary Fig. 5d

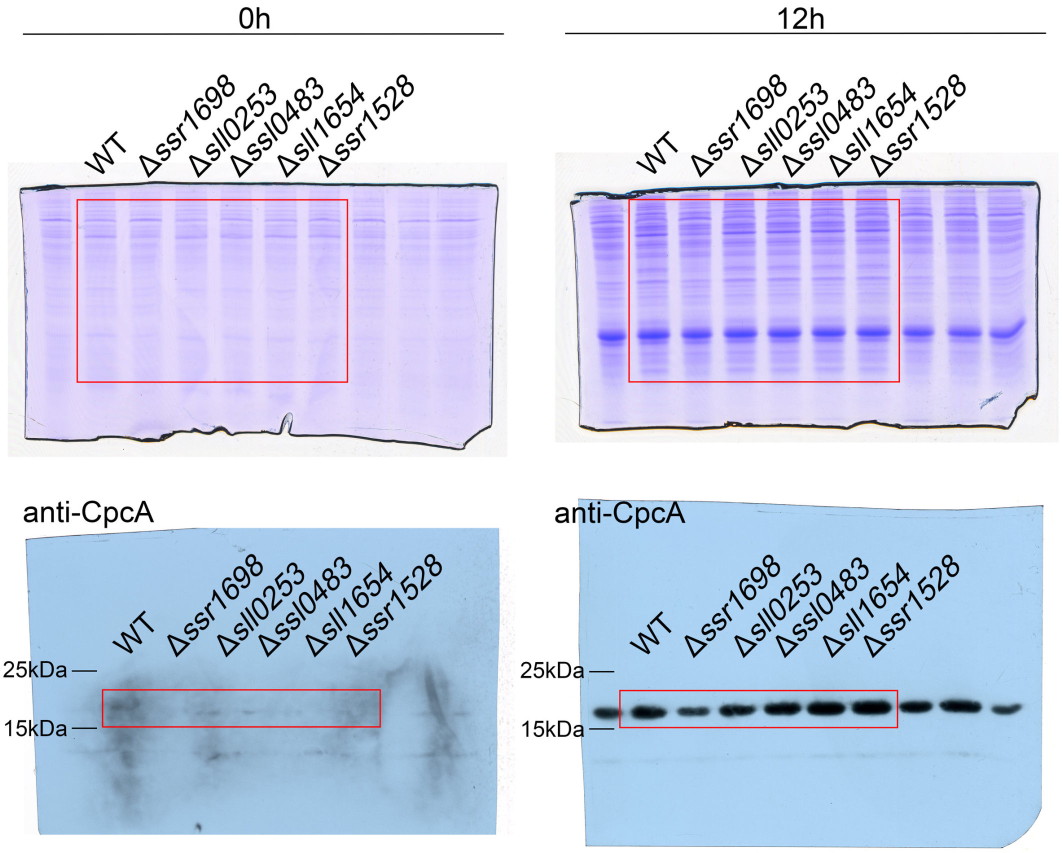

Supplementary Fig. 8b

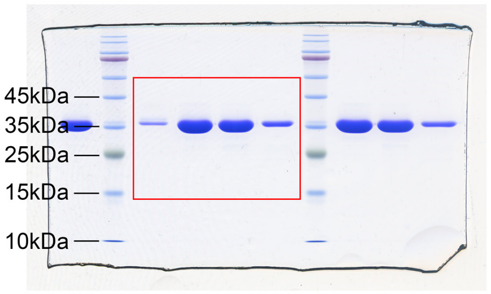

Supplementary Fig. 8c

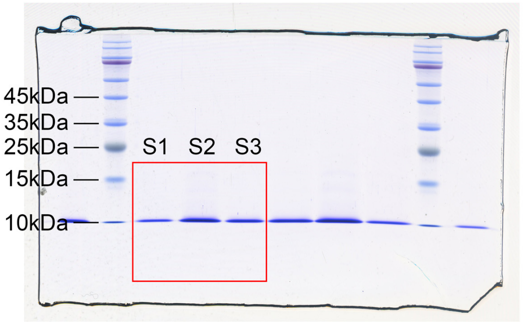

Supplementary Table 1. Primers used in this study.

Primers used to construct the pUC-Δ*ssr1698* vector.

Name	Primer sequence (5'–3')	Purpose
ssr1698-A	GCGTCGACATTGACATCGAAG	Amplification of upstream region
ssr1698-B	GCGGATCCAACCAGAATTCC	
ssr1698-C	CGGGATCCCGTTGTGTCTCAAAATCTC	Amplification of kanamycin gene
ssr1698-D	CGGAGCTCTCTGCCTCGTGAAGAAG	
ssr1698-E	GCGAGCTCTTCTGCCGCTAC	Amplification of downstream region
ssr1698-F	CGGAATTCTTCCGACGAGG	
ssr1698-G	CATTGCTCTAGACGGAATTCTGG	Segregation analysis
ssr1698-H	CGCCTCAAGGCATTGTTAGC	

Primers used to construct the pUC-Δ*sll0253* vector.

Name	Primer sequence (5'–3')	Purpose
sll0253-A	AACTGCAGATTCCAGTTTTCTCACCCCCA	Amplification of upstream region
sll0253-B	GCGGATCCCAATTTTTGCAGAAATTCTTA	
sll0253-C	CGGGATCCCGTTGTGTCTCAAAATCTC	Amplification of kanamycin gene
sll0253-D	CGGAGCTCTCTGCCTCGTGAAGAAG	
sll0253-E	CGGAGCTCGGATAGAGCGATTA	Amplification of downstream region
sll0253-F	CGGAATTCGGTGGCTTCGGTG	
sll0253-G	TTTCTGCAAAAATTGTAACT	Segregation analysis
sll0253-H	ATCCCAAAACTTTCTACCCA	

Primers used to construct the pUC-Δ*ssl0483* vector.

Name	Primer sequence (5'–3')	Purpose
ssl0483-A	GCGTCGACCCATGGACCAATTGGAGTTG	Amplification of upstream region
ssl0483-B	CGGGATCCAAAATTTAATTGCTATGGTT	
ssl0483-C	CGGGATCCCGTTGTGTCTCAAAATCTC	Amplification of kanamycin gene
ssl0483-D	CGGAGCTCTCTGCCTCGTGAAGAAG	
ssl0483-E	CGGAGCTCTGAAACGCTTTTTCTGGT	Amplification of downstream region
ssl0483-F	CGGAATTCTCGTCTTGAAGTTCTCCTG	
ssl0483-G	CAGGCTGTGGCAAAGGGAACCGAGAAT	Segregation analysis
ssl0483-H	ATCAAGTATGCCAAGAAAATCGAGGG	

Primers used to construct the pUC-Δ*sll1654* vector.

Name	Primer sequence (5'–3')	Purpose
sll1654-A	AACTGCAGTACCCTGCGCTTGTCATC	Amplification of upstream region
sll1654-B	GCGGATCCCAAATTTTACAGGCATTG	
sll1654-C	CGGGATCCCGTTGTGTCTCAAAATCTC	Amplification of kanamycin gene
sll1654-D	CGGAGCTCTCTGCCTCGTGAAGAAG	
sll1654-E	CGGAGCTCGGTCAGGGCAAAATTCCCATG	Amplification of downstream region
sll1654-F	CGGAATTCAGGCTCCCAAAACAATCTGCA	
sll1654-G	CGCTGGTTTAATGGGGGTTTTAGTGG	Segregation analysis
sll1654-H	CCAGGGATATAGGTTGGCCATGGTCTTC	

Primers used to construct the pUC-Δ*ssr1528* vector.

Name	Primer sequence (5'–3')	Purpose
ssr1528-A	GCGTCGACTAGTAGATAGGGGAGAA	Amplification of upstream region
ssr1528-B	CGGGATCCGATCAATATTACCCTCGA	
ssr1528-C	CGGGATCCCGTTGTGTCTCAAAATCTC	Amplification of kanamycin gene
ssr1528-D	CGGAGCTCTCTGCCTCGTGAAGAAG	
ssr1528-E	CGAGCTCTCCCATGTTATCTTGA	Amplification of downstream region
ssr1528-F	CGGAATTCTGGCAATCAGGGTAAATT	
ssr1528-G	CTTTTTCGCTAGGATAATTAAAGTAAAATA	Segregation analysis
ssr1528-H	CTTAGTTAGCCAAGAGGCAAGATAGCTAC	

Primers used to construct the pUC-Δ*sll1336* (*argZ*) vector.

Name	Primer sequence (5'–3')	Purpose
ssl1336-A	CGGGATCCTTACCCGCATTGC	Amplification of upstream region
ssl1336-B	CGGGTACCCTGGATGTTAGTCCCC	
ssl1336-C	GGGGTACCAAAATAAAAAAGGGGACCTC	Amplification of spectinomycin gene
ssl1336-D	CGAGCTCAAAATAAAAAAGGGGACCTC	
ssl1336-E	CGGAGCTCCTGGTCGGGATAG	Amplification of downstream region
ssl1336-F	GCGAATTCGGAGTTTCCCCC	
ssl1336-G	CACGATAACCTCGCTTGTGAATC	Segregation analysis
ssl1336-H	GGATTACGCCTGCCTGGAAAG	

Primers used for RT-qPCR.

Name	Primer sequence (5'–3')	Purpose
cpcA-F	CAAACCCAAGGCAACAACTT	*cpcA* transcript
cpcA-R	GTCAAAGGTGCGGTTGATTT	
apcA-F	TGCGCGACATGGACTACTAC	*apcA* transcript
apcA-R	TTCTTTCATTTCCCGGACAC	
cpcG1-F	CCCCCAAAAGTCAAAATGTG	*cpcG1* transcript
cpcG1-R	TTCTCGGTCCCACTTAAACG	
cpcG2-F	CTACTTCCGCAACCAAGCTC	*cpcG2* transcript
cpcG2-R	TAATGCCCCAAGCACTAAGG	
apcE-F	TTTTTGAGCGGGACATTACC	*apcE* transcript
apcE-R	CCAATTCTAGAGCCCTGCTG	
apcF-F	ATCTGCGCTATGCCAGCTAT	*apcF* transcript
apcF-R	CCATGGCTTCAATCATTTCC	
ndhI-F	TCCCATTAACTTGCCCGTAG	*ndhI* transcript
ndhI-R	TAGGCCGCCAATTCATATTC	

Primers used to construct vector to express Ssr1698 protein.

Name	Primer sequence (5'–3')	Purpose
ssr1698-F	CCGGATCCTTATGGCTGATCC	Amplification of *ssr1698* gene
ssr1698-R	GCCTCGAGGCTATTTTTACCAACAC	
